# Genomic Islands as a Marker to Differentiate between Clinical and Environmental *Burkholderia pseudomallei*

**DOI:** 10.1371/journal.pone.0037762

**Published:** 2012-06-01

**Authors:** Thanatchaporn Bartpho, Thidathip Wongsurawat, Surasakdi Wongratanacheewin, Adel M. Talaat, Nitsara Karoonuthaisiri, Rasana W. Sermswan

**Affiliations:** 1 Department of Microbiology, Faculty of Medicine, Khon Kaen University, Khon Kaen, Thailand; 2 Melioidosis Research Center, Khon Kaen University, Khon Kaen, Thailand; 3 National Center for Genetic Engineering and Biotechnology, Pathumthani, Thailand; 4 Department of Animal Health and Biomedical Sciences, University of Wisconsin-Madison, Wisconsin, United States of America; 5 Department of Biochemistry, Faculty of Medicine, Khon Kaen University, Khon Kaen, Thailand; Institut de Pharmacologie et de Biologie Structurale, France

## Abstract

*Burkholderia pseudomallei*, as a saprophytic bacterium that can cause a severe sepsis disease named melioidosis, has preserved several extra genes in its genome for survival. The sequenced genome of the organism showed high diversity contributed mainly from genomic islands (GIs). Comparative genome hybridization (CGH) of 3 clinical and 2 environmental isolates, using whole genome microarrays based on *B. pseudomallei* K96243 genes, revealed a difference in the presence of genomic islands between clinical and environmental isolates. The largest GI, GI8, of *B. pseudomallei* was observed as a 2 sub-GI named GIs8.1 and 8.2 with distinguishable %GC content and unequal presence in the genome. GIs8.1, 8.2 and 15 were found to be more common in clinical isolates. A new GI, GI16c, was detected on chromosome 2. Presences of GIs8.1, 8.2, 15 and 16c were evaluated in 70 environmental and 64 clinical isolates using PCR assays. A combination of GIs8.1 and 16c (positivity of either GI) was detected in 70% of clinical isolates and 11.4% of environmental isolates (P<0.001). Using BALB/c mice model, no significant difference of time to mortality was observed between K96243 isolate and three isolates without GIs under evaluation (P>0.05). Some virulence genes located in the absent GIs and the difference of GIs seems to contribute less to bacterial virulence. The PCR detection of 2 GIs could be used as a cost effective and rapid tool to detect potentially virulent isolates that were contaminated in soil.

## Introduction

*B. pseudomallei* is a Gram-negative bacillus that causes melioidosis. It can be found as an environmental saprophyte in soil or stagnant water in the endemic areas of Southeast Asia and northern Australia [1]. Soil therefore is an important reservoir of the organism. The depth of soils between 15 and 30 cm, with at least 10% of moisture and pH 5–6 were reported to be related to the presence of the organism in soil in northeast Thailand [2]. *B. pseudomallei* is recognized as a Category B agent of bioterrorism by the Centers for Disease Control and Prevention, USA (CDC) [3]. It accounts for 20% of community-acquired septicemias in the northeast of Thailand [4]. N.J. White reported that severe melioidosis cases in Thailand resulted in approximately 50% mortality [5]. Melioidosis presents as a variety of clinical manifestations ranging from acute, sub-acute, chronic, or sub-clinical with the commonest presentation as pneumonia [4]. Three main routes of *B. pseudomallei* infection are ingestion, inhalation and inoculation. Although inhalation is reportedly a common infection route, the actual contribution of each route is unclear [6]. Currently, there is no effective vaccine for melioidosis, and relapse is common and is at an unacceptable rate [6]. Molecular typing methods revealed a large diversity among *B. pseudomallei* isolates, from both environmental and clinical specimens with significant differences in the classification indices between these two sources [7], [8] but isolates from each source can be classified into the same molecular type [6]. Moreover, the clinical and environmental isolates showed no difference in virulence as observed in a mouse model [9]. The genotypes of isolates from each source, however, were not investigated in the study. Nevertheless, the identification of the potentially virulent organism in soil is still important to be used in the control and tracking during an outbreak. Accessory genes from genome analysis organize *B. pseudomallei* into three broad clusters of clinical, environmental and animal groups but overlap is still observed [10], [11]. At present, there is no specific typing method to distinguish the organism in soil as to whether it is saprophytic or a potentially virulent organism.

*B. pseudomallei* K96243 was the first strain whose genome was sequenced and analyzed [12]. As of now, there are several genome sequences of *B. pseudomallei* from various countries submitted to GenBank. Comparative genomic data indicated a high complexity of the genome obtained through horizontal gene acquisition that is an important feature of recent genetic evolution and that has resulted in a genetically diverse pathogenic species [10], [13]. The composition of bacterial genomes can be altered rapidly and dramatically through a variety of processes including horizontal gene transfer (HGT) which incorporates genetic elements from another organism directly into the genome resulting in genomic islands [14]. These sequences can permanently alter bacterial genotypes and result in adaptation to their environment by genome optimization [11]. The term genomic island was introduced to describe regions that contained a diverse range of functions, such as (i) the ability to utilize novel carbon and nitrogen sources (metabolic islands); (ii) the ability to break down novel compounds (degradation islands); (iii) resistance to antibiotic and heavy metals (resistance islands); and (iv) the ability to cause disease (pathogenic islands) [14], [15]. Some pathogens may therefore cause disease by using virulence factors obtained through HGT [16]. Thus, the genomic islands may play an important role in the development of new species, subspecies, and also development of pathotypes [17]. Sixteen GIs were first identified in the *B. pseudomallei* K96243 genome by Holden, et al. [12] and several distinct GIs were depicted from comparative genomes of 5 *B. pseudomallei* strains [13]. GIs 7 and 14 were found to be a part of the bacterial core genome and others may variably contribute at least, in part, to pathogenesis and adaptation to external environments [10]. Analysis of 5 GIs, GI 2, 6, 9, 11 and 16, as representative of various functions in each GI showed high diversity among clinical and environmental isolates and showed no difference between these two sources [18]. Several GIs in *B. pseudomallei* contain metabolic or virulence-related genes that contribute to fitness of the organism and may be selectively present in clinical isolates. These GIs in particular could then be used as a marker to identify potentially virulent organisms in soil used for agriculture or in other environments in the endemic areas.

The whole genome DNA microarray has been used as a tool for genomic comparisons to determine presence or absence of genes in a single hybridization experiment [11]. These arrays provide a powerful method to investigate the plasticity of the genome that may reflect the future bacterial capabilities within a short time. For example, DNA microarrays have been used to investigate genetic contents of closely related species such as *Brucella* spp. [19], *Streptomyces coelicolor* A3 (2) [20], and absence or divergence of *Streptomyces coelicolor* M145 genes in *S. lividans* TK21 [21] or genome plasticity in *Mycobacterium avium* subspecies [22]. The DNA microarray is employed to study the contribution of L-arabinose metabolism to the virulence of *B. pseudomallei* [23]. Comparative genomic hybridization analysis also helps describing the genome evolution through genes lost among *B. pseudomallei*, *B. mallei* and *B. thailandensis* [24], [25].

In this study, the whole genome DNA microarray (*Burkholderia mallei/pseudomallei* microarray) version 2 from the Pathogen Functional Genomics Resource Center was used. The 9,826 oligos, representing all 5,854 ORFs of *B. pseudomallei* K96243, are designed based on ORF sequences across the genomes of *Burkholderia mallei* ATCC 2344, *B. pseudomallei* K96243 and *B. pseudomallei* 1710b to cover as many ORFs of all strains as possible, without unnecessary duplication. The genetic DNA of 2 environmental and 3 clinical isolates were compared with that of *B. pseudomallei* K96243 on the DNA microarray slides to identify regions of genomes that could be used to distinguish clinical from environmental isolates. PCR was subsequently used to confirm the presence of selected regions that distinguish the source of the organism in 70 soil and 64 clinical isolates.

## Results

### Microarray comparison of genomic DNA from five *B. pseudomallei* clinical and environmental isolates with *B. pseudomallei* K96243 strain

**Figure 1:**
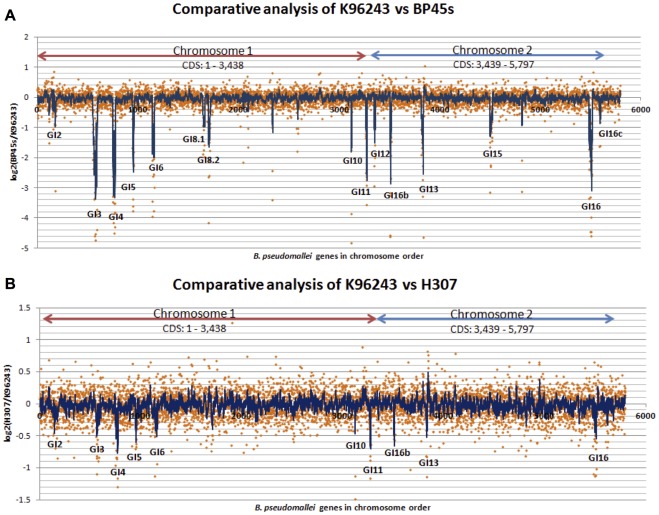
Comparative genome analyses of *B. pseudomallei* K96243 with *B. pseudomallei* from environmental and clinical isolates. The comparison between K96243 and environmental isolate (BP45s) was shown in (A) and clinical isolate (H307) in (B). The log_2_ hybridization ratio of the tested isolate over the K96243 was plotted on the Y axis and positions of genes on the X axis. Low values of the Log_2_ hybridization ratio, seen as peaks, imply absence of genes designated as GIs. The slide averaging window of 6 genes, one gene per step, was applied to the normalized data and smoothed out the fluctuations of the data. Each GI contains at least 6 CDS that have values less than −2SD.

This microarray comparison only allows the detection of regions that are missing in other *B. pseudomallei* isolates relative to the K96243 genome, but could not detect the presence of unique regions of the other isolates. Hybridization results revealed the presence of the majority of K96243 ORFs across the five genomes, as indicated by similar ORFs with signal intensity levels from the microarray comparison (i.e. log_2_ ratios are close to zero; Figure 1). Genes with log_2_ hybridization ratios of at least minus two standard deviations less than the overall mean were considered potentially absent or divergent in the tested isolates. These absent genes when clustered and consisting of at least six consecutive genes were designated as a genomic island (GI). The comparative results between environmental (Figure 1A, BP45s) or clinical isolates (Figure 1B, H307) with K96243 are shown as the log_2_ ratio for each gene position. There were 15 GIs (peaks) detected as absent in five *B. pseudomallei* isolates when compared with the K96243 (Table 1), of which most corresponded to the K96243 GIs (GI2, 3, 4, 5, 6, 8, 10, 11, 12, 13, 15 and 16) reported by Holden et al. [12] and the GI16b as reported by Tuanyok et al. [13]. The largest GI, GI8 (92.3 kb), in the K96243 strain was seen as two separated absent regions of 15.7 kb (CDS coordinates BPSL1638-BPSL1656) and 21.8kb (CDS coordinates BPSL1693-1708A) with distinguishable %GC contents (Table 1). They were assigned here as GIs8.1 and 8.2 because they were located at the same reference genomic location with GI8 in K96243 but contained different gene contents. Since the methods used here compared gene compositions with the K96243, it can only report regions present in or absent from the K96243, but cannot identify the unique gene composition in other isolates. Therefore, the GI nomenclature as proposed by Tuanyok et al. [13] may not be properly applied. There were four conserved GIs (GIs1, 7, 9 and 14) present in all 5 isolates, of which GIs7 and 9 were reported to be a part of the core genome of *B. pseudomallei* strain [12].

**Table 1:** Summary of the genomic islands (GIs) identified to be absent in tested isolates when compared with GIs in the K96243.

GI	Coordinates of absent region		Size	No.	%GC
K96243	start CDS	stop CDS	(kb)	CDSs	
GI 2	BPSL0140	BPSL0176	27.3	37	65.7
GI 3	BPSL0549A	BPSL0588	46.6	43	56.7
GI 4	BPSL0745	BPSL0770	36.5	26	56.4
GI 5	BPSL0939	BPSL0953	20.5	15	57.5
GI 6	BPSL1137	BPSL1157	14.6	22	58.9
GI 8.1*	BPSL1638	BPSL1656	15.7	17	61.6
GI 8.2*	BPSL1693	BPSL1708A	21.8	16	59.0
GI 10	BPSL3113	BPSL3118	7.1	6	54.6
GI 11	BPSL3257	BPSL3269	12.7	13	55.8
GI 12	BPSL3342	BPSL3349	9.5	8	56.8
GI 16b**	BPSS0068	BPSS0080	16.7	13	59.4
GI 13	BPSS0378	BPSS0391A	18.4	18	59.0
GI 15	BPSS1047	BPSS1089	35.1	50	65.3
GI 16	BPSS2046	BPSS2076	50.4	34	60.8
GI 16c***	BPSS2148	BPSS2154	8.3	8	69.5

CDS = protein coding sequence.

* Absent region identified in GI8 of K96243 to contain 2 sub-GIs.

** A new GI discovered in *B. pseudomallei* genome (Tuanyok et al., 2008).

*** A new GI detected by DNA microarray in this study.

Comparative genome hybridization in this study also detected a new GI, named GI16c located downstream from GI16 on chromosome 2, with the size of 8.3 kb determined by the microarray. It consists of eight contiguous probes (CDS coordinates BPSS2148-BPSS2154) containing common characteristics of a GI such as a transposase gene and insertion element. It appeared to be absent in all soil isolates and present in 1/3 of tested clinical isolates.

Moreover, from the log_2_ hybridization ratios of genes from each isolate to those of a clinical isolate of the reference K96243, higher similarity (shorter peaks, closer to 0) was observed in each GI in the clinical isolates than in the soil isolates (Figure 1). The overall profiles from the comparison of environmental isolates (2 total) and clinical isolates (3 total) to the K96243 were also different (Figure S1). When the presence or absence of each GI in these 5 isolates was compared with K96243, clinical isolates (H307, P54, P82) contained lower numbers of absent genomic islands (10–11 absent GIs) than environmental isolates (BP45s and BP28L). GIs 6, 12 and 16c in clinical isolates showed some variability in the GI patterns. A total of eight regions (GIs2, 3, 4, 5, 11, 13, 16 and 16b) were absent in both tested clinical and soil isolates. GIs 8.1, 8.2 and 15 were absent in the soil isolates but present in all of the clinical isolates. Their functions are related to metabolism, cellular process, biosynthesis and transport proteins (Table 2).

**Table 2:** The GIs determined by DNA microarrays and their functional classification.

	*B. pseudomallei* isolates					
GIs	Soil		Clinical			Function classification*
	BP45s	BP28L	H307	P54	P82	
2	−	−	−	−	−	Cellular processes, Regulatory, Cell envelope, DNA metabolism,
						Transcription
3	−	−	−	−	−	Regulatory, Cell envelope, DNA metabolism, Protein fate, Energy
						metabolism
4	−	−	−	−	−	Cellular processes/ Energy metabolism, Regulatory, DNA
						metabolism
5	−	−	−	−	−	DNA metabolism, Transport and binding protein
6	−	−	−	+	+	Cell envelope, Energy metabolism/protein synthesis/regulatory
8.1	−	−	+	+	+	Energy metabolism, Regulatory, Central metabolism, Cell
						envelope, Transport and binding protein
8.2	−	−	+	+	+	DNA metabolism, Regulatory, Cellular processes, Fatty acid
10	−	+	−	−	−	DNA metabolism, Regulatory
11	−	−	−	−	−	DNA metabolism
12	−	−	+	−	−	Cellular protein, Protein fate
16b	−	−	−	−	−	Cell envelope, Regulatory, Transport and binding protein
13	−	−	−	−	−	DNA metabolism, Regulatory, Transcription
15	−	−	+	+	+	Cellular processes/biosynthesis, Regulatory, Cell envelope, DNA
						metabolism, Transcription, Energy metabolism
16	−	−	−	−	−	Cellular process/protein fate, Energy metabolism/AA biosynthesis,
						Regulatory, Central metabolism, Transcription, Transport and binding protein
16c	−	−	+	−	−	Biosynthesis/transport, Transport protein

GIs present (+) and absent (−).

* Functional classification data obtained by *Burkholderia pseudomallei* K96243 genome annotation from Pathema Bioinformatics Resource Center [28].

The absence of the GIs3, 4, 6, 10, 13, 15 and 16c identified by DNA microarray was selected for further confirmation by PCR amplification with primers flanking a gene outside (positive control) and a gene inside each GI. The data correlated with the microarray data (data not shown).

### PCR detection of selected GIs to differentiate clinical and environmental isolates

GIs 8.1, 8.2 and 15 found only in 3 clinical but not in 2 soil isolates used in CGH and a new GI (GI16c) discovered here were selected and validated by PCR in 70 environmental and 64 clinical isolates. The GI 13 absent in all 5 tested isolates was included as a negative control. The percentage of positive detections of GI8.1, 8.2, 15 and 16c was 60.9, 32.8, 9.4 and 50% in clinical isolates and 10, 2.9, 5.7 and 4.3% in soil isolates (Table 3). The presence of GI13, a negative control, was rarely found in only 2% of all samples. These results support the findings obtained from DNA microarray data. Moreover, when these GIs were used to differentiate clinical isolates from soil isolates by PCR detection, GI8.1, 8.2 and 16c but not GI15 gave significantly different results (P<0.001). GIs8.1 and 16c were commonly present in 39 clinical isolates and other 6 isolates were positive for GI16c. Therefore, the PCR detection of GI8.1 and 16c in combination (positivity of either GI) as markers can detect 70% (45/64) of clinical isolates and 11.4% (8/70) of environmental isolates (P<0.001) (Table 3).

**Table 3:** PCR detection of four GIs in 70 soil and 64 clinical *B. pseudomallei* isolates.

GIs	GI patterns					% Positive by PCR		P-value
	Soil		Clinical			Soil	Clinical	
	BP45s	BP28L	H307	P54	P82	(n = 70)	(n = 64)	
8.1	−	−	+	+	+	10	60.9	<0.001
8.2	−	−	+	+	+	2.9	32.8	<0.001
15	−	−	+	+	+	5.7	9.4	0.518
16c	−	−	+	−	−	4.3	50	<0.001
8.1+16c	−	−	+	+	+	11.4	70.3	<0.001
8.1+8.2+16c	−	−	+	+	+	11.4	75	<0.001

### Association between genomic islands and bacterial virulence in mice

The virulence of BP45S (ribotype 23) with 15 GIs absent, P54 (ribotype 23) and P82 (ribotype 13) (Table 4) with 11 GIs absent when compared with K96243 and the referenced K96243 strain were evaluated in BALB/c mice with intraperitonealy injection doses of 2×10^6^, 1×10^5^ and 5×10^3^ cfu. The highest dose gave less than 30% survival of all groups within 3 days and the lowest dose prolonged survival more than 90% in all groups for more than 30 days. Only 1×10^5^ cfu showed a difference in survival rates. Repeated evaluation of survival rates in other independent experiments with 1×10^5^ cfu injections, however, did not show a significant difference of survival rates between clinical and environmental isolates (P = 0.6) or between each isolate and K96243 (data not shown).

**Table 4:** *B. pseudomallei* isolates used for DNA microarray.

Strain	Host	Sample origin	Ribotype
BP45S	Environment	Soil	23
BP28L	Environment	Soil	13
H307	Male, 54 years	Blood	23
P54	Female, 50 years	Pus	23
P82	Male, 56 years	Pus	13

## Discussion

Comparative genome hybridization of five tested isolates to K96243 indicated their average core genome close to 86% as reported by Sim et al. [10]. The microarray-based CGH technology has been shown as a remarkable tool for the identification and fine discrimination between close species, and additionally provided insight into the adaptation to its ecological niche as reported in *L. taiwanensis* BL263 [26]. The variability of GIs among clinical isolates was higher than that of environmental isolates. The different overall comparison profiles between clinical and environmental isolates, as shown in figure 1 and figure S1 of the other 3 isolates can clearly distinguish the source of isolation. Even though the transfer of GIs is assumed to be spontaneous, stress responses may provide selective pressure responsible for variation of GIs and gene insertion in each GI of the clinical isolates may play an important role for host adaptation. The absence or divergence of *B. pseudomallei* GIs found by comparative genome hybridization using the K96243 sequenced strain as a reference was consistent with the recent studies using bioinformatics tools or *in silico* analysis [12], [13], [18]. Moreover, two out of four GIs (GsI7 and 9) found to be common among the 5 tested isolates are also reported as parts of the core genome by Sim et al.[10]. Interestingly, the method was able to identify a new GI of the genomic islands (GI 16c) on chromosome 2, which was not previously reported [12], [13]. A small difference in %GC (69.5%) of GI16c when compared to the rest of the *B. pseudomallei* genome (68%) and a lack of flanking tRNA and 3′ end repeat may make it difficult to be detected by other genome sequence comparison methods. GI16c, however, contains transpose and insertion element genes which are involved in genome mobility and are common characteristics of GIs. This GI may be in the stage of evolutionary regression that eliminated some GI functions [27].

The GI 8, which is the largest GI of K96243, was identified in this study to contain 2 absent regions with a distinguishable %GC. The percentage of these 2 regions present in 64 clinical isolates was also clearly different. This GI region was reported to contain several types of gene contents that varied in size (15.8–92.3 kb) [13]. The hyper-variation in this location supported the cause of genome plasticity created by various GIs. The comparative genome analysis of the *B. pseudomallei* genomes available in the public domain [28] also indicated a common finding of variability in the number of genes and gene shuffling within each GI obtained through horizontal transfer from other organisms.

The PCR detection for the presence of five GIs, GIs2, 6, 9, 11 and 16, with various functions, among 186 environmental and clinical isolates was reported to be no different [18]. Two environmental isolates in this study were carefully picked from an undisturbed soil location in Nampong district, Khon Kaen province as soil representative isolates. When they were used together with 3 clinical isolates in comparative genome hybridization, 3 GIs (GIs8.1, 8.2 and 15) were clinically specific GIs as they were only identified in clinical but not soil isolates. Their functions are related to metabolism and transport proteins that may support the survival of the organism inside their host. The PCR detection of these GIs indicated their low presentation in soil isolates (3–10%) but varied among clinical isolates. Interestingly, the new GI16c was rarely detected in soil samples but was present in up to 50% in clinical isolates. GIs8.1, 8.2 and 16c can significantly distinguish clinical from soil isolates. The PCR detection using a combination of primers specific to GIs8.1 and 16c was positive up to 70% of clinical isolates. The access of *B. pseudomallei* genomes through the VISTA component of Integrated Microbial Genomes (IMG) [29] was done to investigate the presence of these 2 GIs (GIs8.1 and 16c) in 22 publically available genome sequences, of which 4 are complete and 18 are drafts [30]. Twenty-one of them are clinical isolates from several countries including Thailand and 1 is an environmental isolate (S13) from Singapore. GIs8.1 and 16c were present in 57 (12/21) and 62 (13/21)% of clinical isolates and 71.4% by 2 GIs detection. None of them were present in the S13 soil isolate [28]. A combination of the 3 primer sets for GIs8.1, 8.2 and 16c aids the percentage of clinical isolate detections in this study to 76.6% (Table 3). Therefore, the development of multiplex PCR for GI8.1 and 16c detection or all these 3 GIs can be applied to detect potentially virulent isolates in soil in endemic areas of northeast Thailand in a single process. As GIs in each *B. pseudomallei* isolate are diverse and may be changed through adaptation and evolution, further evaluation of this PCR detection with isolates from other areas is therefore advised before it can be applied to other regions of the world.

In this study, it is clear that differences of time to mortality were not observed in BALB/c mice model after inoculation with *B. pseudomallei* clinical and environmental isolates with the same ribotype (ribotype 23) and another clinical isolate of ribotype 13 [8] with a different absence of GIs. Clinically specific GIs encode several genes with metabolic and transport functions that might be important in generation and acquisition of nutrients. Examples of these genes are the ABC transport system, hydrolase, oxygenase and the Gnt-R family of regulatory proteins. The islands also include genes encoded for the outer membrane porin protein and a surface exposed protein such as BPSL1705, a Yad A- like protein (adhesion gene of *Yersinia* sp.). These genes were suggested to promote the virulence of *B. pseudomallei* through mediating host-cell interactions and therefore were previously suggested to be important in bacterial survival or pathogenesis [12], [31]. The BPSL1705 gene, however, was confirmed by PCR amplification to be absent in all tested isolates but was still present in the *B. pseudomallei* K96243 (data not shown). Therefore, these genes might not play a direct role in the virulence of this bacterium as previously suggested, or there are some other alternatives genes with similar functions to this gene in other isolates. A similar story was also found for the cell surface protein or hemagglutinin-related protein gene (BPSS2053) (in GI 16) that was absent in all tested isolates but present in the K96243. This gene was proposed to play a role in virulence by reducing the adherence to human buccal epithelial cells [10]. Therefore, the difference in severity of the disease should depend at least on a combination of genes with more influence from host's status as seen by the high risk of this disease in diabetic people. Nevertheless, the possibility that the absent GI may be associated with acquisition of the bacteria that were omitted by direct inoculation or the low sample size or even simply that mice are different from humans has to be considered before it can be concluded that the virulence of each isolate is not different.

Besides examining the virulence genes located in some absent GIs, PCR was used to investigate the presence of genes outside the GIs that encode for other known virulence determinants such as type III protein secretion systems (*bsaQ*), that are potential virulence determinants such as surface polysaccharides (BPSL2800), fimbriae and pili (BPSS0120) [12]. They were all present in all 5 tested isolates. This may suggest that these other virulence factors present in the *B. pseudomallei* genome are critical to maintain its virulence. Similarly, regulatory genes known to promote survival such as a sigma factor (rpoS) [32] were present in all isolates. These factors in *B. pseudomallei* may contribute to the fitness of the organism as well or even more than those obtained recently from horizontal gene transfer.

In addition, the microarray data analysis of all isolates revealed the presence of other virulence genes such as types I, II, III and VI protein secretion systems [33], exoproteins and genes known to promote survival in diverse and challenging environments including secondary metabolite biosynthesis genes (siderophore, malleobactin biosynthesis cluster, superoxide detoxification, Ser proteases, phospholipase C, catalase, etc.) [12], [34], [35], [36], [37], sigma factor [32], [38], quorum sensing [39], reactive nitrogen intermediates [40], O-antigen [41], chaperonin GroEL [42], multidrug effect pump, secondary metabolism functions or drug resistance functions, intracellular stress and motility and chemotaxis [12]. Therefore, these data confirm the previous report from Ulett et al, 2001 that there is no difference in virulence between clinical and environmental isolates. As their work does not analyze the genotype of the compared isolates, this study could then provide additional details to summarize that the outcome of the infection mainly depends on the immune response or susceptibility of the host rather than the genetic background of the bacteria.

In conclusion, through whole genome comparative analysis by DNA microarray hybridization, a new GI (GI16c) present on chromosome 2 of *B. pseudomallei* was identified. The technique was also able to show different hybridization profiles between clinical and environmental isolates and identify 3 GIs that were found more commonly in clinical isolates. The use of PCR to detect GI8.1 and GI16c is a simple and cost effective method to screen for potentially virulent isolates in the environment in endemic areas, and it can further be applied for pathophysiological studies of the bacteria.

## Materials and Methods

### Strains and culture conditions

*B. pseudomallei* K96243 sequenced strain was isolated from a patient in Khon Kaen hospital, Thailand [12]. BP45s, BP28L and the other 70 environmental isolates were isolated from undisturbed soil in 3 districts of Khon Kaen province, northeast Thailand (Table S1). H307, P54, P82 and the other 64 clinical isolates were isolated from patients admitted to the Srinagarind hospital, Khon Kaen province and other 16 provincial hospitals in the northeast Thailand (Table S2). Details of the five isolates used in CGH are summarized in Table 4. Ribotyping is a typing system based on the diversity of ribosomal RNA gene of the organism [8] and was applied to distinguish the ribotypes between environmental and clinical isolates to be used in CGH. The bacteria were identified by biochemical tests and confirmed by latex agglutination with a monoclonal antibody specific to *B. pseudomallei* [43]. The requirement for approval the use of all clinical isolates in a study of this nature is exempt according to the human ethics committee, Khon Kaen University.

To prepare a *B. pseudomallei* culture for mouse infection, 1% of an overnight culture of each bacterium in trypticase soy broth (TSB) was sub-cultured into TSB and incubated at 37^°^C with shaking until the OD600nm reached the mid-log phase. By using the growth curve for each isolate, each bacterium was diluted with phosphate buffered saline (pH 7.2) to the desired number of cells that was later determined exactly by plate count.

### Genomic DNA extraction and labeling

The overnight culture of *B. pseudomallei* in Luria Bertani broth (LB broth) was used for genomic DNA extraction according to the manufacturer's protocol (Generation capture column, QIAGEN Sciences, MD, USA). The 10–15 µg genomic DNA of *B. pseudomallei* K96243 used as reference strain was labeled with Cy3-dCTP whereas each *B. pseudomallei* isolate tested (BP45s, BP28L, H307, P54, P82) was labeled with Cy5-dCTP fluorescent dye using a standard nick-translation reaction to generate randomly labeled DNA fragments according to the manufacturer's protocol (Promega, Madison, WI, USA). After the labeling reaction was completed, unincorporated nucleotides were removed by passage over a gel filtration spin column (Illustra MicroSpin™ G-50 Columns, GE Healthcare Bio-Sciences, NJ, USA) according to the manufacturer's recommendations.

### DNA microarray

The whole genome DNA microarray slides of *B. mallei* and *B. pseudomallei* version 2.0 were received from the PFGRC (Pathogen Functional Genomics Research Center, MD, USA). The array contains a total of 9,826 oligo probes (70-mer) representing multiple strains and/or species (5,440 ORFs of *B. mallei*, 5,854 ORFs of *B. pseudomallei* K96243 and 6,348 ORFs of *B. pseudomallei* 1710b). Each probe was spotted in duplicate. Details of the *Burkholderia mallei/pseudomallei* microarray were published through the Pathogen Functional Genomics Research Center (PFGRC) [44].

### Microarray hybridization and image analysis

The standard protocols established by the PFGRC for slide preparation and hybridization were used [44]. Briefly, the array slides were immersed in the prehybridization solution (5x SSC, 0.1% SDS, 1% BSA) at 42°C for at least one hour. The slides were washed with MilliQ water until suds disappeared and washing was continued twice more for 2 min each with MilliQ water on a rotary shaker. The slides were then washed with isopropyl alcohol for 2 min before centrifuging at 1,000×*g* for 10 min at room temperature.

For genomic DNA comparison, the DNA probes were prepared by mixing 2 µg each of Cy5 labeled gDNA of the K96243 and Cy3 labeled gDNA of a selected isolate and then drying in a SpeedVac concentrator (SPD1010 SpeedVac System, Thermo Savant, USA) for about 30 min. The probe was re-suspended in 50 µl hybridization solution (50% formamide, 5x SSC, 0.1% SDS) and hybridized onto the post-processed slides while the LifterSlip Microarray Coverslips (Erie Scientific, USA) were placed over the array slides and incubated at 42°C in a water bath for 16–20 h. Each slide was then washed according to the standard protocols recommended by PFGRC.

### Microarray data analysis

The hybridized slides were scanned with a GenePix 4000B laser scanner (Molecular Devices, Sunnyvale, CA, USA) and initially analyzed with GenePix Pro 6.1 software. Briefly, signals below the threshold level (the total pixel intensity of the spot is 65% lower than the median background intensity at both wavelengths) or “empty” (ORF without oligo) and “bad” (flagged by visual inspection of the spot images) spots were removed before normalization. The data were normalized within each array by the scaled print-tip (Lowess) method [45], [46] and across arrays using the Aroma package [47], [48] available from Centre for Mathematical Sciences, Lund, Sweden [49], using R project environment [50]. The analysis for the presence or absence of GIs was observed based on K96243 ORFs on the microarray. The low values of Log_2_ hybridization ratios imply absence of genes visualized by Treeview software was provided as figure S2. The CDS coordinates of genes in 15 GIs were listed as table S3. The microarray data have been deposited in NCBIs Gene Expression Omnibus [51] and all data is MIAME compliant which are accessible through GEO Series accession number GSE19558 [52] for comparative genomics hybridizations.

### GC content analysis of GIs

To calculate %GC content of each GI, a Python computer script was written following a method of Madigan et al. [53] by using complete nucleotide sequences of *B. pseudomallei* K96243 chromosomes 1 and 2 (downloaded from NCBI, GenBank: NC_006350.1 and NC_006351.1) as the input data.

### PCR verification of microarray data

The presence or absence of GIs3, 4, 6, 10, 13 and 16c in 5 tested isolates from the microarray hybridization was confirmed using primers flanking a gene outside (positive control) and a gene inside each GI by using TaqDNA polymerase (Roche Applied Science, Indianapolis, IN). Further confirmation of the presence of some selected virulence genes; *FliC*, *bsaQ*, *rpoS*, BPSL2800, BPSS0120, BPSL1705 and BPSS2053 was also performed. Primers for amplification of each GI and important genes were listed in table S4. The presence of GIs8.1, 8.2, 13, 15 and 16c were detected in 70 environmental and 64 clinical *B. pseudomallei* isolates. The nucleotide sequences of primers to detect 5 GIs are listed in Table 5.

**Table 5:** Primer sequences used to detect 5 GIs in all *B. pseudomallei* isolates (139 total).

Gene	Primers
GI8.1 (BPSL1642)	Forward AGTGCTAAGGCACCTGGAAA
	Reverse GCGGGAAAGATCCTCCTTAT
GI8.2 (BPSL1708)	Forward AACCCCTCACAACGAAAGG
	Reverse GCCGCTGATTCCTGAGATAC
GI13 (BPSS0379)	Forward CTACGCTTGCGCTTGTCTC
	Reverse CCGAGCGAGTTTATCTCCAG
GI15 (BPSS1057)	Forward CCAGTTGCTCGATGACCATA
	Reverse CCGAGTTGGTGAACGTCAG
GI16c (BPSS2152)	Forward CTCGTCTATGCGTACGATGC
	Reverse CCAGCCGAACACCAGATAGT

### Virulence of *B. pseudomallei* isolates in BALB/c mice

BALB/c mice (all male or female, 4 to 6 weeks of age) were obtained from the National Laboratory Animal Center, Mahidol University, Bangkok, Thailand. The maintenance and care of the experimental animals complied with the National Animal Center guidelines. The *B. pseudomallei* infected BALB/c mouse protocol was approved by the animal ethics committee, Khon Kaen University, Khon Kaen, Thailand (Reference No. 0514.1.12.2/2). The most suitable numbers of bacteria were titrated to obtain the killing efficiency that could be observed within 30 days; almost all of the infected mice died. The experiment was performed separately in duplicate to confirm the reliability of the results. Therefore, groups of eight BALB/c mice, one group at a time, were injected intraperitoneally with 100 µl of PBS containing a suitable number of selected bacteria used in the microarray experiment. The control group was injected with PBS. The survival of the mice was recorded for 30 days post-infection.

### Statistical analysis

The differences in detection for the presence of GIs8.1, 8.2, 13, 15 and 16c by PCR between the clinical and environmental groups were tested using chi-square. Survival times of mice infected with various *B. pseudomallei* isolates were compared using Kaplan-Meier curves and the log-rank test. Data were considered statistically significant at P<0.05 (GraphPad Prism 5.0 software).

## Supporting Information

Figure S1 Comparative genome analysis of *B. pseudomallei* K96243 with *B. pseudomallei* BP28L environmental isolate (A), clinical isolates P54 (B) and P82 (C). Low values of Log_2_ hybridization ratio imply absence of genes designated as GIs from 1–10 in chromosomes I and 11–15 in chromosome II. A slide averaging window of 6 genes, one gene per step, was applied to the normalized data and smoothed out the fluctuations of the data. Each GI contains at least 6 CDS that have the value less than −2SD.
(DOC)

Figure S2 **Data from microarray of all 15 absent GIs in** ***B. pseudomallei*** **isolates.** Low values of Log_2_ hybridization ratios imply absence of genes visualized by Treeview software.
(DOC)

Table S1 **The result of PCR detection of 4 GIs in 70 soil isolates collected from 3 districts in Khon Kaen province, northeast, Thailand.**
(PDF)

Table S2 **The result of PCR detection in 64 clinical isolates obtained from melioidosis patients in 17 hospitals, northeast, Thailand.**
(PDF)

Table S3 **CDSs coordinates of genes in 15 GIs.**
(DOC)

Table S4 **Primer sequences used for GIs confirmation and specific genes amplification.**
(DOC)
