# Supplementary figures and images for: Genomic Islands as a Marker to Differentiate between Clinical and Environmental Burkholderia pseudomallei
Source: PLoS One. 2012 Jun 1;7(6):e37762. doi: 10.1371/journal.pone.0037762 (PMC3365882; doi:10.1371/journal.pone.0037762)

**
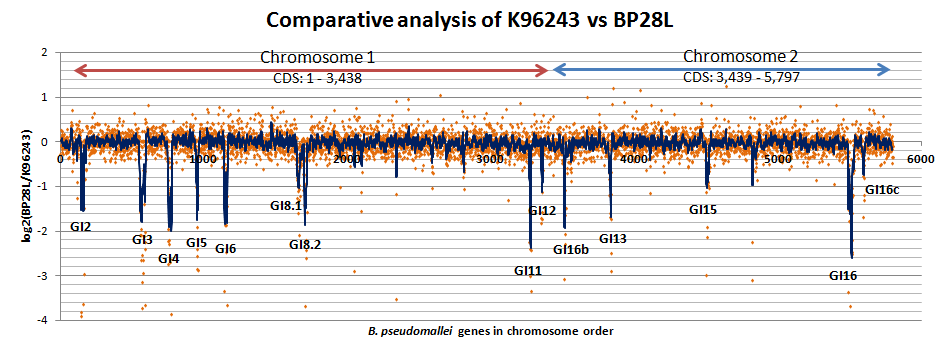
**

A

B

**
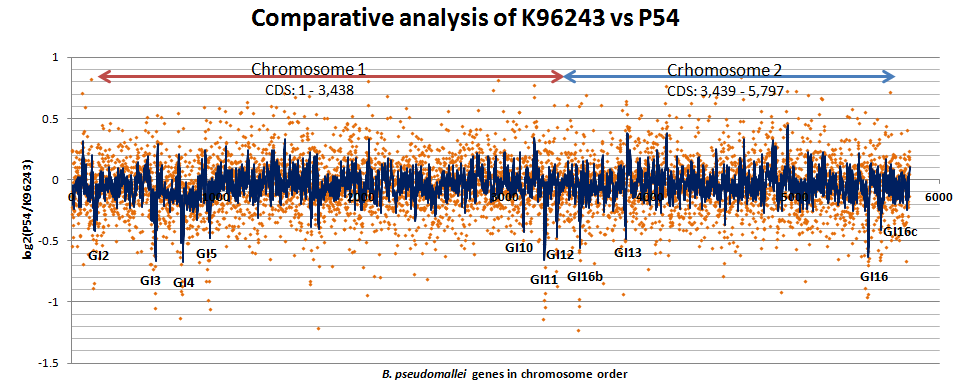
**

C

**
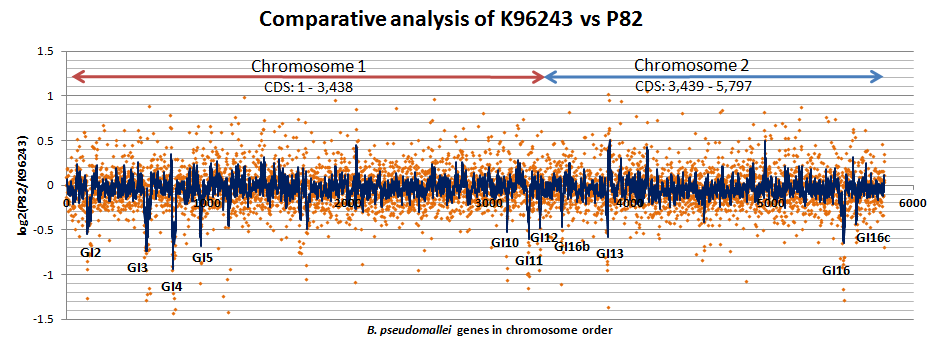
**

**Figure S1**

Supplement: Figure S1 — Comparative genome analysis of B. pseudomallei K96243 with B. pseudomallei BP28L environmental isolate (A), clinical isolates P54 (B) and P82 (C). Low values of Log2 hybridization ratio imply absence of genes designated as GIs from 1–10 in chromosomes I and 11–15 in chromosome II. A slide averaging window of 6 genes, one gene per step, was applied to the normalized data and smoothed out the fluctuations of the data. Each GI contains at least 6 CDS that have the value less than −2SD. (DOC) [file pone.0037762.s001.doc]
