# Supplementary material for: Genomic Islands as a Marker to Differentiate between Clinical and Environmental Burkholderia pseudomallei
Source: PLoS One. 2012 Jun 1;7(6):e37762. doi: 10.1371/journal.pone.0037762 (PMC3365882; doi:10.1371/journal.pone.0037762)
Supplement: Figure S2 — Data from microarray of all 15 absent GIs in B. pseudomallei isolates. Low values of Log2 hybridization ratios imply absence of genes visualized by Treeview software. (DOC) [file pone.0037762.s002.doc]

**Figure S2**

**GI 2 (BPSL0140-0176)**


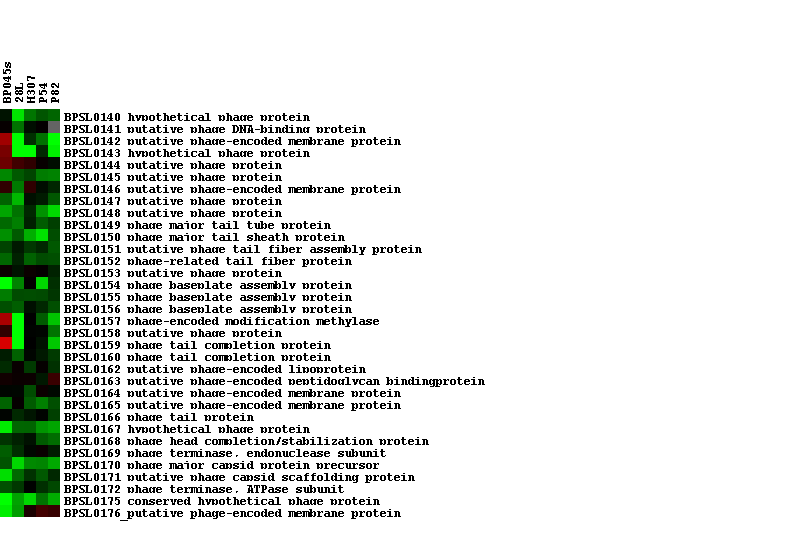


**GI 3 (BPSL0549A-0588)**

**GI 4 (BPSL0745-0770)**

**GI 5 (BPSL0939-0953)**

**GI 6 (BPSL1137-1157)**

**GI 8.1 (BPSL1638-1656)**

**GI 8.2 (BPSL1693-1708A)**

**
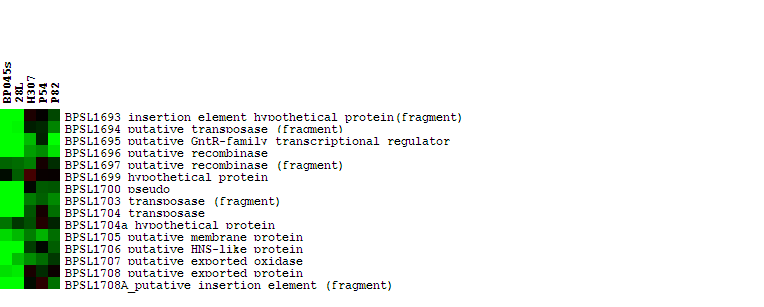
**

**GI 10 (BPSL3113-3118)**

**
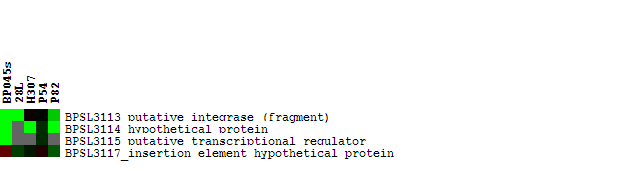
**

**GI 11 (BPSL3257-3269)**

**GI 12 (BPSL3342-3349)**

**GI 16b (BPSS0068-0080)**

**
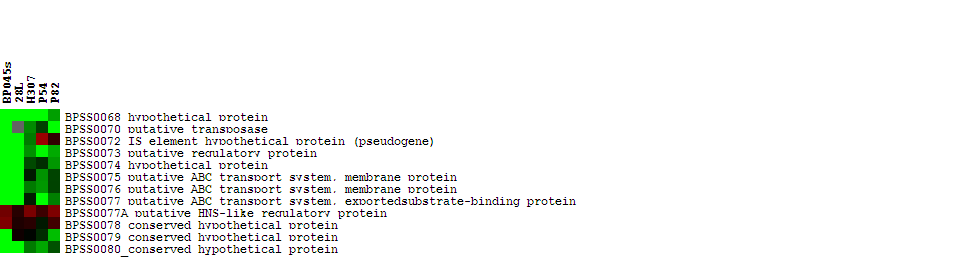
**

**GI 13 (BPSS0378-0391A)**

**GI 15 (BPSS1047-1089)**

**GI 16 (BPSS2046-2076)**

**GI 16c (BPSS2148-2154)**

**
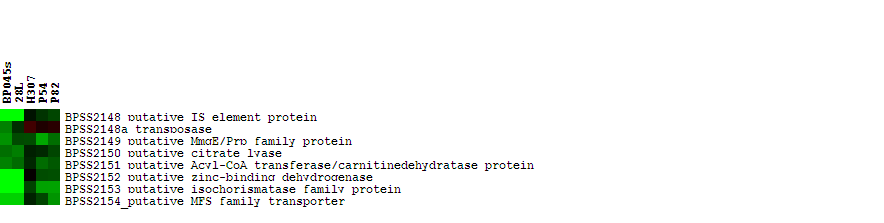
**
