# Supplementary material for: Genomic Islands as a Marker to Differentiate between Clinical and Environmental Burkholderia pseudomallei
Source: PLoS One. 2012 Jun 1;7(6):e37762. doi: 10.1371/journal.pone.0037762 (PMC3365882; doi:10.1371/journal.pone.0037762)
Supplement: Table S1 — The result of PCR detection of 4 GIs in 70 soil isolates collected from 3 districts in Khon Kaen province, northeast, Thailand. (PDF) [file pone.0037762.s003.pdf]

| Soil isolates | Location (districts in Khon Kaen province) | GI8.1 | GI8.2 | GI15 | GI 16c |
|---|---|---|---|---|---|
| BP003 | Nam Pong | - | - | - | - |
| BP004 | Nam Pong | - | - | - | - |
| BP005 | Nam Pong | - | - | - | - |
| BP007 | Nam Pong | - | - | - | - |
| BP008 | Nam Pong | - | - | - | - |
| BP010 | Nam Pong | - | - | - | + |
| BP011 | Nam Pong | - | - | - | - |
| BP013 | Nam Pong | + | - | - | - |
| BP020L | Nam Pong | - | - | - | - |
| BP020S | Nam Pong | - | - | - | - |
| BP021S | Nam Pong | - | - | - | - |
| BP021L | Nam Pong | - | - | - | - |
| BP022S | Nam Pong | - | - | - | - |
| BP024L | Nam Pong | - | - | - | - |
| BP024S | Nam Pong | - | - | - | - |
| BP030L | Nam Pong | - | - | - | - |
| BP036L | Nam Pong | - | - | - | - |
| BP037S | Nam Pong | - | - | - | - |
| BP039S | Nam Pong | - | - | - | - |
| BP039L | Nam Pong | - | - | - | - |
| BP040S | Nam Pong | - | - | - | - |
| BP040L | Nam Pong | - | - | - | - |
| BP041S | Nam Pong | - | - | - | - |
| BP043S | Nam Pong | - | - | - | - |
| BP044L | Nam Pong | - | - | - | - |
| BP047L | Nam Pong | - | - | - | - |
| BP048L | Nam Pong | - | - | - | - |
| BP050L | Nam Pong | - | - | - | - |
| BP051 | Nam Pong | - | - | - | - |
| BP052 | Nam Pong | - | - | - | - |
| BP054 | Nam Pong | - | - | - | - |
| BP057 | Nam Pong | - | - | - | - |
| BP059 | Nam Pong | - | - | - | - |
| BP060 | Nam Pong | - | - | - | - |
| BP062 | Nam Pong | - | - | - | - |
| BP063 | Nam Pong | - | - | - | - |
| BP064 | Nam Pong | - | - | - | - |
| BP065 | Nam Pong | - | - | - | - |
| BP066 | Nam Pong | - | - | - | - |
| BP68-2 | Nam Pong | - | - | - | - |
| BP69 | Nam Pong | - | - | - | - |
| BP71 | Nam Pong | - | - | - | - |
| BP72 | Nam Pong | - | - | - | - |
| BP82-1 | Nam Pong | - | - | - | - |
| BP86-1 | Nam Pong | - | - | - | - |
| BP88 | Nam Pong | - | - | - | - |

| | | | | | |
|---|---|---|---|---|---|
| BP047S | Nam Pong | - | - | - | - |
| BP053 | Nam Pong | - | - | - | - |
| KKU 1-1 | Muang | + | + | - | + |
| KKU 1-2 | Muang | + | + | - | - |
| KKU 2-1 | Muang | - | - | + | - |
| KKU 2-2 | Muang | - | - | - | - |
| KKU 3-4 | Muang | + | - | - | - |
| KKU 4-2 | Muang | - | - | - | - |
| KKU 5-2 | Muang | - | - | - | - |
| KKU 6-1 | Muang | - | - | - | - |
| KKU 7-5 | Muang | - | - | - | - |
| KKU 8-2 | Muang | - | - | - | - |
| KKU 9-2 | Muang | - | - | + | - |
| KKU 10-1 | Muang | + | - | - | + |
| KKU 11-3 | Muang | - | - | - | - |
| KKU 12-3 | Muang | - | - | - | - |
| KKU 13-1 | Muang | - | - | - | - |
| KKU 14-2 | Muang | - | - | - | - |
| KKU 15-1 | Muang | - | - | - | - |
| KKU 16-2 | Muang | - | - | + | - |
| N1 | Khao Suan Kwang | - | - | - | - |
| N4 | Khao Suan Kwang | + | - | + | - |
| N9 | Khao Suan Kwang | + | - | - | - |
| N13 | Khao Suan Kwang | - | - | - | - |
