# Supplementary material for: Genomic Islands as a Marker to Differentiate between Clinical and Environmental Burkholderia pseudomallei
Source: PLoS One. 2012 Jun 1;7(6):e37762. doi: 10.1371/journal.pone.0037762 (PMC3365882; doi:10.1371/journal.pone.0037762)
Supplement: Table S2 — The result of PCR detection in 64 clinical isolates obtained from melioidosis patients in 17 hospitals, northeast, Thailand. (PDF) [file pone.0037762.s004.pdf]

Clinical isolates	Specimens	Location of hospital (province)	GI8.1	GI8.2	GI15	GI16c
EPBR052	pus	BURIRUN	-	-	-	-
H140	hemoculture	CHAIYAPUM	-	+	+	+
H568	hemoculture	CHAIYAPUM	-	-	-	-
H84	hemoculture	CHAIYAPUM	-	-	-	-
H574	hemoculture	CHAIYAPUM	+	+	-	+
P35	pus	KALASIN	-	-	-	-
P37	hemoculture	KALASIN	+	-	+	-
2-119	urine	KHON KAEN	+	+	-	+
2-148	urine	KHON KAEN	+	-	-	+
2-166	urine	KHON KAEN	-	+	-	-
2-371	urine	KHON KAEN	+	+	-	-
2-390	urine	KHON KAEN	+	-	-	+
2-494	urine	KHON KAEN	-	-	-	+
2-861	urine	KHON KAEN	-	+	+	+
4-121	fluid	KHON KAEN	+	+	-	-
4-131	fluid	KHON KAEN	-	-	-	-
4-132	fluid	KHON KAEN	+	-	-	+
4-218	fluid	KHON KAEN	+	-	-	+
4-44	fluid	KHON KAEN	+	-	-	+
P83	pus	KHON KAEN	+	-	+	+
SP07	sputum	KHON KAEN	+	+	-	+
SP19	sputum	KHON KAEN	+	-	-	-
SP248	sputum	KHON KAEN	+	+	-	+
SP310	sputum	KHON KAEN	+	-	-	-
SP77	sputum	KHON KAEN	+	-	-	-
U173	urine	KHON KAEN	+	-	+	+
U389	urine	KHON KAEN	-	-	-	-
FL144	fluid	KHON KAEN	+	-	-	-
FL202	fluid	KHON KAEN	-	-	-	-
EPKKU12	pus	KHON KAEN	+	-	-	-
EPKKU13	pus	KHON KAEN	+	-	-	+
EPKKU19	fluid	KHON KAEN	+	-	-	+
EPKKU27	sputum	KHON KAEN	-	-	-	-
EPKKU28	hemoculture	KHON KAEN	+	-	+	+
EPKKU6	pus	KHON KAEN	+	+	-	-
EPKKU7	hemoculture	KHON KAEN	-	-	-	-
EPKKU9	skull	KHON KAEN	-	-	-	+
P009	pus	MAHASALAKAM	-	-	-	-
P475	pus	MAHASALAKAM	+	-	-	+
EPMK152	hemoculture	MAHASALAKAM	+	+	-	+
H20	hemoculture	MUKDAHAN	+	-	-	-
EPMD078	hemoculture	MUKDAHAN	+	+	-	+
EPMD077	hemoculture	MUKDAHAN	-	-	-	-

EPNP001	pus	NAKHONPANOM	-	-	-	+
P171	pus	NAKORNRATCHASIMA	+	+	-	+
P263	pus	NAKORNRATCHASIMA	-	-	-	-
EPNK013	fluid	NONG KHAI	+	+	-	+
P69	pus	NONGBUALUMPU	-	-	-	-
EPRE062	hemoculture	ROI-ET	+	+	-	+
EPSN213	sputum	SAKHON NAKHON	-	-	-	+
EPSN223	urine	SAKHON NAKHON	+	-	-	+
EPSN032	pus	SAKHON NAKHON	-	-	-	-
EPSK002	hemoculture	SRI SA KED	+	-	-	+
1026b	hemoculture	UBONRATCHATHANI	+	+	-	+
1901A	sputum	UBONRATCHATHANI	+	-	-	-
EPUT005	pus	UDONTHANI	+	+	-	-
EPUT044	pus	UDONTHANI	-	+	-	-
EPUT179	sputum	UDONTHANI	+	-	-	+
EPUT457	hemoculture	UDONTHANI	-	+	-	-
EPUT017	hemoculture	UDONTHANI	+	+	-	-
EPAC006	hemoculture	UMNARDCHAROEN	+	-	-	+
EPAC012	pus	UMNARDCHAROEN	-	-	-	-
EPAC019	hemoculture	UMNARDCHAROEN	+	+	-	+
EPYT066	sputum	YASOTHORN	-	-	-	-
