# Supplementary material for: Genomic Islands as a Marker to Differentiate between Clinical and Environmental Burkholderia pseudomallei
Source: PLoS One. 2012 Jun 1;7(6):e37762. doi: 10.1371/journal.pone.0037762 (PMC3365882; doi:10.1371/journal.pone.0037762)
Supplement: Table S3 — CDSs coordinates of genes in 15 GIs. (DOC) [file pone.0037762.s005.doc]

| **Table S3** | |
| --- | --- |
| **CDS coordinates of 15 genomic islands** | |
| **ID** | **Product** |
| **GI 2** |  |
| BPSL0140 | hypothetical protein |
| BPSL0141 | putative phage DNA-binding protein |
| BPSL0142 | putative phage-encoded membrane protein |
| BPSL0143 | hypothetical protein |
| BPSL0144 | hypothetical protein |
| BPSL0145 | putative phage protein |
| BPSL0146 | putative phage-encoded membrane protein |
| BPSL0147 | putative phage protein |
| BPSL0148 | putative phage protein |
| BPSL0149 | phage major tail tube protein |
| BPSL0150 | phage major tail sheath protein |
| BPSL0151 | putative phage tail fiber assembly protein |
| BPSL0152 | phage-related tail fiber protein |
| BPSL0153 | putative phage protein |
| BPSL0154 | phage baseplate assembly protein |
| BPSL0155 | phage baseplate assembly protein |
| BPSL0156 | phage baseplate assembly protein |
| BPSL0157 | phage-encoded modification methylase |
| BPSL0158 | putative phage protein |
| BPSL0159 | phage tail completion protein |
| BPSL0160 | phage tail completion protein |
| BPSL0161 | putative phage protein |
| BPSL0162 | putative phage-encoded lipoprotein |
| BPSL0163 | putative phage-encoded peptidoglycan bindingprotein |
| BPSL0164 | putative phage-encoded membrane protein |
| BPSL0165 | putative phage-encoded membrane protein |
| BPSL0166 | phage tail protein |
| BPSL0167 | hypothetical phage protein |
| BPSL0168 | phage head completion/stabilization protein |
| BPSL0169 | phage terminase, endonuclease subunit |
| BPSL0170 | phage major capsid protein precursor |
| BPSL0171 | putative phage capsid scaffolding protein |
| BPSL0172 | phage terminase, ATPase subunit |
| BPSL0173 | putative phage portal vertex protein |
| BPSL0174 | putative phage DNA-binding protein |
| BPSL0175 | conserved hypothetical phage protein |
| BPSL0176 | putative phage-encoded membrane protein |
| **GI 3** |  |
| BPSL0549A | putative DNA-binding protein |
| BPSL0550 | hypothetical protein |
| BPSL0551 | hypothetical protein |
| BPSL0552 | hypothetical protein |
| BPSL0553 | putative DNA-binding protein |
| BPSL0554 | hypothetical phage protein |
| BPSL0555 | putative membrane protein |
| BPSL0556 | hypothetical protein |
| BPSL0557 | hypothetical protein |
| BPSL0557A | putative phage protein |
| BPSL0558 | putative DNA-binding protein |
| BPSL0559 | hypothetical protein |
| BPSL0560 | hypothetical protein |
| BPSL0561 | putative exported protein |
| BPSL0562 | putative DNA-binding protein |
| BPSL0563 | hypothetical protein |
| BPSL0564 | hypothetical protein |
| BPSL0565 | hypothetical protein |
| BPSL0566 | hypothetical protein |
| BPSL0567 | hypothetical protein |
| BPSL0568 | hypothetical protein |
| BPSL0569 | conserved hypothetical protein |
| BPSL0570 | conserved hypothetical protein |
| BPSL0571 | putative membrane protein |
| BPSL0572 | hypothetical protein |
| BPSL0573 | putative exported protein |
| BPSL0574 | subtilase family protein |
| BPSL0574A | hypothetical phage protein |
| BPSL0574B | hypothetical protein |
| BPSL0575 | hypothetical protein |
| BPSL0576 | hypothetical protein |
| BPSL0577 | phage integrase family protein |
| BPSL0578 | dienelactone hydrolase family protein |
| BPSL0579 | hypothetical protein |
| BPSL0580 | hypothetical protein |
| BPSL0581 | conserved hypothetical protein |
| BPSL0582 | hypothetical protein |
| BPSL0583 | hypothetical protein |
| BPSL0584 | putative membrane protein |
| BPSL0585 | hypothetical protein |
| BPSL0586 | hypothetical protein |
| BPSL0587 | phage integrase family protein |
| BPSL0588 | hypothetical protein |
| **GI 4** |  |
| BPSL0745 | hypothetical protein |
| BPSL0746 | hypothetical protein |
| BPSL0747 | hypothetical protein |
| BPSL0747a | hypothetical protein |
| BPSL0748 | hypothetical protein |
| BPSL0749 | hypothetical protein |
| BPSL0750 | hypothetical protein |
| BPSL0751 | hypothetical protein |
| BPSL0752 | hypothetical protein |
| BPSL0753 | hypothetical protein |
| BPSL0754 | putative integrase/recombinase(fragment) |
| BPSL0756 | putative DNA-binding protein |
| BPSL0757 | hypothetical protein |
| BPSL0758 | putative phosphoesterase |
| BPSL0759 | hypothetical protein |
| BPSL0760 | hypothetical protein |
| BPSL0761 | hypothetical protein |
| BPSL0762 | putative RNA 2"-phosphotransferase |
| BPSL0763 | putative helicase SNF2 family protein |
| BPSL0764 | hypothetical protein |
| BPSL0765 | putative helicase family protein |
| BPSL0766 | hypothetical protein |
| BPSL0767 | putative phospholipase protein |
| BPSL0768 | conserved hypothetical protein |
| BPSL0769 | hypothetical protein |
| BPSL0770 | conserved hypothetical protein |
| **GI 5** |  |
| BPSL0939 | putative DeoR family regulatory protein |
| BPSL0940 | conserved hypothetical protein |
| BPSL0941 | hypothetical protein |
| BPSL0942 | hypothetical protein |
| BPSL0943 | putative insertion element protein |
| BPSL0944 | putative phage integrase/recombinase protein |
| BPSL0945 | conserved hypothetical protein |
| BPSL0946 | conserved hypothetical protein |
| BPSL0947 | putative type I restriction enzyme specificityprotein |
| BPSL0948 | putative type I restriction-modificationmethylase |
| BPSL0949 | hypothetical protein |
| BPSL0950 | insertion element hypothetical protein |
| BPSL0951 | pseudo |
| BPSL0952 | putative replication protein |
| BPSL0953 | hypothetical protein |
| **GI 6** |  |
| BPSL1137 | hypothetical protein |
| BPSL1138 | hypothetical protein |
| BPSL1139 | hypothetical protein |
| BPSL1140 | putative phage-related protein |
| BPSL1141 | hypothetical protein |
| BPSL1142 | putative phage-related protein |
| BPSL1143 | putative phage terminase |
| BPSL1144 | hypothetical protein |
| BPSL1145 | putative phage-related protein |
| BPSL1146 | hypothetical protein |
| BPSL1147 | hypothetical protein |
| BPSL1148 | hypothetical protein |
| BPSL1149 | hypothetical protein |
| BPSL1150 | hypothetical protein |
| BPSL1151 | hypothetical protein |
| BPSL1152 | hypothetical protein |
| BPSL1153 | hypothetical protein |
| BPSL1153A | hypothetical protein |
| BPSL1154 | hypothetical protein |
| BPSL1155 | hypothetical protein |
| BPSL1156 | hypothetical protein |
| BPSL1157 | putative phage integrase |
| **GI 8.1** |  |
| BPSL1638 | putative phage-related protein (fragment) |
| BPSL1639 | putative transposase(fragment) |
| BPSL1642 | putative GntR-family regulatory protein |
| BPSL1643 | conserved hypothetical protein |
| BPSL1644 | putative hydrolase |
| BPSL1645 | putative oxygenase |
| BPSL1646 | putative monooxygenase |
| BPSL1647 | putative betaine aldehyde dehydrogenase |
| BPSL1648 | conserved hypothetical protein |
| BPSL1649 | putative ABC transport system, substrate-bindingprotein |
| BPSL1650 | putative ABC transport system, permease protein |
| BPSL1651 | putative ABC transport system, permease protein |
| BPSL1652 | putative ABC transport system, ATP-bindingprotein |
| BPSL1653 | putative GntR-family regulatory protein |
| BPSL1654 | succinate-semialdehyde dehydrogenase [NADP+] |
| BPSL1655 | putative outer membrane porin protein |
| BPSL1656 | hypothetical protein |
| **GI 8.2** |  |
| BPSL1693 | insertion element hypothetical protein(fragment) |
| BPSL1694 | putative transposase(fragment) |
| BPSL1695 | putative GntR-family transcriptional regulator |
| BPSL1696 | putative recombinase |
| BPSL1697 | putative recombinase(fragment) |
| BPSL1699 | hypothetical protein |
| BPSL1700 | pseudo |
| BPSL1702 | putative invertase |
| BPSL1703 | transposase(fragment) |
| BPSL1704 | transposase |
| BPSL1704a | hypothetical protein |
| BPSL1705 | putative membrane protein |
| BPSL1706 | putative HNS-like protein |
| BPSL1707 | putative exported oxidase |
| BPSL1708 | putative exported protein |
| BPSL1708A | pseudo |
| **GI 10** |  |
| BPSL3113 | putative integrase(fragment) |
| BPSL3114 | hypothetical protein |
| BPSL3115 | putative transcriptional regulator |
| BPSL3116 | putative transposase |
| BPSL3117 | insertion element hypothetical protein |
| BPSL3118 | putative restriction modification system methylase |
| **GI 11** |  |
| BPSL3257 | putative plasmid recombinase |
| BPSL3258 | conserved hypothetical protein |
| BPSL3259 | putative plasmid conjugal transfer protein |
| BPSL3260 | conserved hypothetical protein |
| BPSL3261 | hypothetical protein |
| BPSL3262 | putative plasmid conjugal transfer protein |
| BPSL3263 | putative plasmid conjugal transfer protein |
| BPSL3264 | putative plasmid conjugal transfer protein |
| BPSL3265 | putative plasmid conjugal transfer protein |
| BPSL3266 | hypothetical protein |
| BPSL3267 | hypothetical protein |
| BPSL3268 | putative membrane protein |
| BPSL3269 | hypothetical protein |
| **GI 12** |  |
| BPSL3342 | putative bacteriophage protein |
| BPSL3343 | putative bacteriophage protein |
| BPSL3344 | putative bacteriophage integrase |
| BPSL3345 | putative bacteriophage-related protein |
| BPSL3346 | hypothetical protein |
| BPSL3347 | putative bacteriophage-related lipoprotein |
| BPSL3348 | putative bacteriophage protein |
| BPSL3349 | putative membrane protein |
| **GI 16b** |  |
| BPSS0068 | hypothetical protein |
| BPSS0069 | transposase(pseudogene) |
| BPSS0070 | putative transposase |
| BPSS0072 | IS element hypothetical protein (pseudogene) |
| BPSS0072A | conserved hypothetical protein (fragment) |
| BPSS0073 | putative regulatory protein |
| BPSS0074 | hypothetical protein |
| BPSS0075 | putative ABC transport system, membrane protein |
| BPSS0076 | putative ABC transport system, membrane protein |
| BPSS0077 | putative ABC transport system, exportedsubstrate-binding protein |
| BPSS0078 | hypothetical protein |
| BPSS0079 | hypothetical protein |
| BPSS0080 | hypothetical protein |
| **GI 13** |  |
| BPSS0378 | putative phage integrase |
| BPSS0379 | hypothetical protein |
| BPSS0380 | putative DNA-binding regulatory protein |
| BPSS0380A | hypothetical protein |
| BPSS0380B | hypothetical protein |
| BPSS0381 | putative DNA-binding regulatory protein |
| BPSS0382 | hypothetical protein |
| BPSS0383 | putative DNA-binding protein |
| BPSS0384 | hypothetical protein |
| BPSS0384A | hypothetical protein |
| BPSS0385 | hypothetical protein |
| BPSS0386 | transposon Tn2501 resolvase |
| BPSS0387 | putative phage-related protein |
| BPSS0388 | putative phage-related protein |
| BPSS0389 | putative phage-related protein |
| BPSS0390 | conserved hypothetical protein |
| BPSS0391 | putative phage-related hypothetical protein |
| BPSS0391A | hypothetical protein |
| **GI 15** |  |
| BPSS1047 | hypothetical bacteriophage protein |
| BPSS1047a | hypothetical bacteriophage protein |
| BPSS1048 | hypothetical bacteriophage protein |
| BPSS1048a | hypothetical bacteriophage protein |
| BPSS1048b | hypothetical bacteriophage protein |
| BPSS1048c | hypothetical bacteriophage protein |
| BPSS1048d | hypothetical bacteriophage protein |
| BPSS1049 | hypothetical bacteriophage protein |
| BPSS1050 | hypothetical bacteriophage protein |
| BPSS1051 | hypothetical bacteriophage protein |
| BPSS1052 | hypothetical bacteriophage replication protein |
| BPSS1053 | hypothetical bacteriophage-acquired protein |
| BPSS1054 | hypothetical bacteriophage-acquired protein |
| BPSS1055 | putative partition protein |
| BPSS1056 | copG family protein |
| BPSS1057 | putative bacteriophage gp29 protein |
| BPSS1058 | putative bacteriophage gp30 protein |
| BPSS1059 | putative bacteriophage gp31 protein |
| BPSS1060 | hypothetical bacteriophage protein |
| BPSS1061 | putative DNA binding protein |
| BPSS1062 | putative phage protein |
| BPSS1063 | bacteriophage terminase, ATPase subunit |
| BPSS1064 | putative bacteriophage protein |
| BPSS1065 | putative major capsid protein precursor |
| BPSS1066 | putative bacteriophage terminase, endonucleasesubunit |
| BPSS1067 | putative bacteriophage headcompletion/stabilization protein |
| BPSS1068 | hypothetical bacteriophage protein |
| BPSS1069 | putative bacteriophage tail protein X |
| BPSS1070 | putative bacteriophage membrane protein |
| BPSS1071 | putative bacteriophage membrane protein |
| BPSS1072 | bacteriophage-acquired protein |
| BPSS1073 | putative bacteriophage protein |
| BPSS1073A | putative phage protein |
| BPSS1074 | bacteriophage tail completion protein R |
| BPSS1075 | bacteriophage tail completion protein S |
| BPSS1076 | hypothetical bacteriophage protein |
| BPSS1077 | site-specific DNA methyltransferase |
| BPSS1078 | putative bacteriophage baseplate assemblyprotein V |
| BPSS1079 | putative bacteriophage baseplate assemblyprotein W |
| BPSS1080 | bacteriophage baseplate assembly protein J |
| BPSS1081 | putative bacteriophage tail protein I |
| BPSS1082 | putative bacteriophage protein gp17 |
| BPSS1083 | putative bacteriophage-acquired protein |
| BPSS1084 | bacteriophage major tail sheath protein |
| BPSS1085 | putative bacteriophage major tail tube protein |
| BPSS1086 | putative bacteriophage protein |
| BPSS1086a | hypothetical phage protein |
| BPSS1087 | bacteriophage membrane protein |
| BPSS1088 | putative bacteriophage tail-related protein |
| BPSS1089 | bacteriophage late control gene D protein |
| **GI 16** |  |
| BPSS2046 | putative IS element transposase |
| BPSS2047 | chrolohydrolase |
| BPSS2048 | glutathione S-transferase |
| BPSS2049 | putative IS element transposase |
| BPSS2050 | pseudo |
| BPSS2051 | DNA-binding protein |
| BPSS2052 | activator/secretion protein |
| BPSS2053 | cell surface protein |
| BPSS2054 | conserved hypothetical protein |
| BPSS2055 | hypothetical protein |
| BPSS2056 | hypothetical protein |
| BPSS2057 | IS element transposase |
| BPSS2058 | putative ATP-binding inner membrane transportprotein |
| BPSS2059 | conserved hypothetical protein |
| BPSS2060 | L-asparaginase |
| BPSS2061 | conserved hypothetical protein |
| BPSS2061A | putative transposase(fragment) |
| BPSS2062 | acetyltransferase(GNAT) family protein |
| BPSS2063 | hypothetical protein |
| BPSS2063a | hypothetical protein |
| BPSS2064 | putative porin protein |
| BPSS2065 | putative fatty aldehyde dehydrogenase |
| BPSS2066 | conserved hypothetical protein |
| BPSS2067 | putative aldose 1-epimerase |
| BPSS2068 | short chain dehydrogenase |
| BPSS2069 | ABC transporter, ATP-binding protein |
| BPSS2070 | branched-chain amino acid transport systempermease |
| BPSS2071 | putative exported protein |
| BPSS2072 | mandelateracemase/ muconatelactonizingenzyme |
| BPSS2073 | GntR family regulator protein |
| BPSS2074 | senescence marker protein-30 (SMP-30) familyprotein |
| BPSS2074a | pseudo |
| BPSS2075 | hypothetical protein |
| BPSS2076 | pseudo |
| **GI 16c** |  |
| BPSS2148 | putative IS element protein |
| BPSS2148a | transposase |
| BPSS2149 | putative MmgE/Prp family protein |
| BPSS2150 | putative citrate lyase |
| BPSS2151 | putative Acyl-CoA transferase/carnitinedehydratase protein |
| BPSS2152 | putative zinc-binding dehydrogenase |
| BPSS2153 | putative isochorismatase family protein |
| BPSS2154 | putative MFS family transporter |
