# Supplementary material for: Genomic Islands as a Marker to Differentiate between Clinical and Environmental Burkholderia pseudomallei
Source: PLoS One. 2012 Jun 1;7(6):e37762. doi: 10.1371/journal.pone.0037762 (PMC3365882; doi:10.1371/journal.pone.0037762)
Supplement: Table S4 — Primer sequences used for GIs confirmation and specific genes amplification. (DOC) [file pone.0037762.s006.doc]

**Table S4**

**Primers for amplify region**

GI3 (BPSL0552-BPSL0557) Product size

BPSL0551_Forward GGTGCCATGATGCTATTCG 4.5 Kb

BPSL0557A_Reverse GAGTCAGGCGCAAGAAGAGT

GI3 (BPSL0571-BPSL0572)

BPSL0571_Forward ACAATCGGAAATTGCTTGCT 2.7 Kb

BPSL0573_Reverse GGGCTCATAACTGGTTGCAT

GI4 (BPSL0744-BPSL0745)

BPSL0743_Forward GATGAGAAGGGAATGGACGA 3.8 Kb

BPSL0746_Reverse GTAGCAGTGCCCCTCGATAA

GI6 (BPSL1150-BPSL1154)

BPSL1149_Forward GCGAGTCCGAATGGAATGTA 2.1 Kb

BPSL1155_Reverse AGCGCTCGTCTCTTTCGAT

GI8.2 (BPSL1693-1708A)

BPSL1692_Forward GTACCGCCCTGAACGAACT 23 Kb

BPSL1709_Reverse AGCAACGACGAAACCAGATG

GI10 (BPSL3112-BPSL3117)

BPSL3111_Forward ATCTGCTGTACGACGGCTTC 7.7 Kb

BPSL3118_Reverse GGTGCCATGATGCTATTCG

GI13 (BPSS0388-BPSS0391)

BPSS0387_Forward ACTTTGCAGGACGGAACACT 5 Kb

BPSS0392_Reverse CACGATCCAGAGCATGAGTC

GI15 (BPSS1047-BPSS1048)

BPSS1046_Forward ATCCGTACAGCCACTCGAAC 2.8 Kb

BPSS1048c_Reverse AGTGGTCGCAATTCCTTCAT

GI16c (BPSS2147-BPSS2149)

BPSS2146_ Forward TTCAGGTGCATCACGTTCAT 4 Kb

BPSS2150_Reverse GACCGTCTATCACACGCTGA

**Primers for specific genes within GI**

BPSL0146 putative phage-encoded membrane protein Product size

Forward GAAGCTAAGAAGGCGCAGAA 140 bp

Reverse CGCATCATGGTCAGATTGTC

BPSL0551 hypothetical protein

Forward GACAAGGATGTGAGGGCCTA 100 bp

Reverse CCACATTCGGACTGACAATG

BPSL0554 hypothetical protein

Forward GAGAACGACTTGTGCGTCTG 108 bp

Reverse GAGAACGACTTGTGCGTCTG

BPSL0561 hypothetical protein

Forward CTTGTGTCCGCTCAGAATCA 205 bp

Reverse AGTATTCGCCGTCGTGAAGT

BPSL0566 hypothetical protein

Forward AACCATCATGGACCTTCTCG 231 bp

Reverse CGCTTGAAGGGATACAGCTC

BPSL0571 hypothetical protein

Forward ACAATCGGAAATTGCTTGCT 232 bp

Reverse GACGGTGGATTCTGCGTTAT

BPSL0574b hypothetical protein

Forward AGGAAAGCGAGCTACCATCA 112 bp

Reverse GGGCATCTCTTACCTCATCG

BPSL0576 hypothetical protein

Forward CCATCGATTGTCACGTTTTG 387 bp

Reverse TATGCTCGCAGCTGTATTGG

BPSL0581 hypothetical protein

Forward TATGCTCGCAGCTGTATTGG 180 bp

Reverse GGATCCATTTCGTCTTCGAC

BPSL0583 hypothetical protein

Forward CCAGTTTGAATTCCGACGTT 131 bp

Reverse TGACATCGTAGCGGTCTTTG

BPSL0585 hypothetical protein

Forward AGATAGAGCCGGACGCATAA 155 bp

Reverse AGATAGAGCCGGACGCATAA

BPSL0746 hypothetical protein

Forward TTGGGGATTACCATGTACGG 207 bp

Reverse GTAGCAGTGCCCCTCGATAA

BPSL0757 hypothetical protein

Forward CAGGTGCCAGAAAGCTGATT 250 bp

Reverse GTGACCTCCAACTGGCTTGT

BPSL0941 hypothetical protein

Forward ATGCGCAAGGACTTTGAACT 185 bp

Reverse CGGTTTCGGGAATAGTCGTA

BPSL1149 hypothetical protein

Forward GCTGACATGGCCGAGTAGTT 149 bp

Reverse GCGAGTCCGAATGGAATGTA

BPSL1705 hypothetical protein

Forward GCTGTGGCTGGTACGGTAAT 224 bp

Reverse GGATCGGTGGTCTGTGAAGT

BPSL3260 hypothetical protein

Forward TGTCGTGGCCCGGGGATTTGTA 238 bp

Reverse TATTCGTTGCTTTCGCGTGTGGTC

BPSS0387 hypothetical protein

Forward ACCCAGTACCCGTTGATCTG 116 bp

Reverse ACTTTGCAGGACGGAACACT

BPSS1055 partition protein

Forward GATGACATGGCGAAATTGCT 248 bp

Reverse TCGATTTCTGCACACGCTAC

BPSS2053 cell surface protein

Forward TTGCATCGTTGTCGAAGAAG 200 bp

Reverse CCCAATCGATGTTCCAGACT

**Primers for important genes**

BPSS2053 cell surface protein

Forward TTGCATCGTTGTCGAAGAAG 200 bp

Reverse CCCAATCGATGTTCCAGACT

BPSL1705 hypothetical protein

Forward GCTGTGGCTGGTACGGTAAT 224 bp

Reverse GGATCGGTGGTCTGTGAAGT
